# Multi-scale reservoir characterization of pre-rift reservoirs in the October field, Gulf of Suez using integrated seismic, well logs, and core data

**DOI:** 10.1038/s41598-025-27530-8

**Published:** 2025-12-05

**Authors:** Mahmoud Ghaly, Adel Ali Ali Othman, Mohammed Elkhawaga, Taher Mostafa

**Affiliations:** 1 Faculty of Science, Geology Department, Al-Azhar University, P.O. Box 11884, Nasr City, Cairo, Egypt; 2 Gulf of Suez Petroleum Company, Cairo, Egypt

**Keywords:** Gulf of Suez Rift Basin, Multi-scale reservoir characterization, Hydraulic flow units, Flow zone indicator, Energy science and technology, Solid Earth sciences

## Abstract

This study presents an integrated multi-scale characterization of pre-rift reservoirs in the October Field, Gulf of Suez, to resolve critical uncertainties in compartmentalization and resource potential. By synergizing 2D seismic, well logs, and core data, we delineate a structural framework dominated by NW–SE and NE-SW fault systems that compartmentalize the reservoirs. Our analysis establishes the superior reservoir potential of the Nubia Formation compared to the Matulla Formation. A key novelty of this work is the definition of five distinct Hydraulic Flow Units (HFUs) through the integration of Flow Zone Indicator (FZI) and Stratigraphic Modified Lorenz (SML) methods. This provides a robust, core-calibrated quantitative framework for predicting permeability in uncored intervals, significantly reducing prediction uncertainty. The workflow successfully identifies high-flow-efficiency units and optimal exploration targets, demonstrating a transferable approach for re-evaluating mature rift basins.

## Introduction

**Fig. 1:**
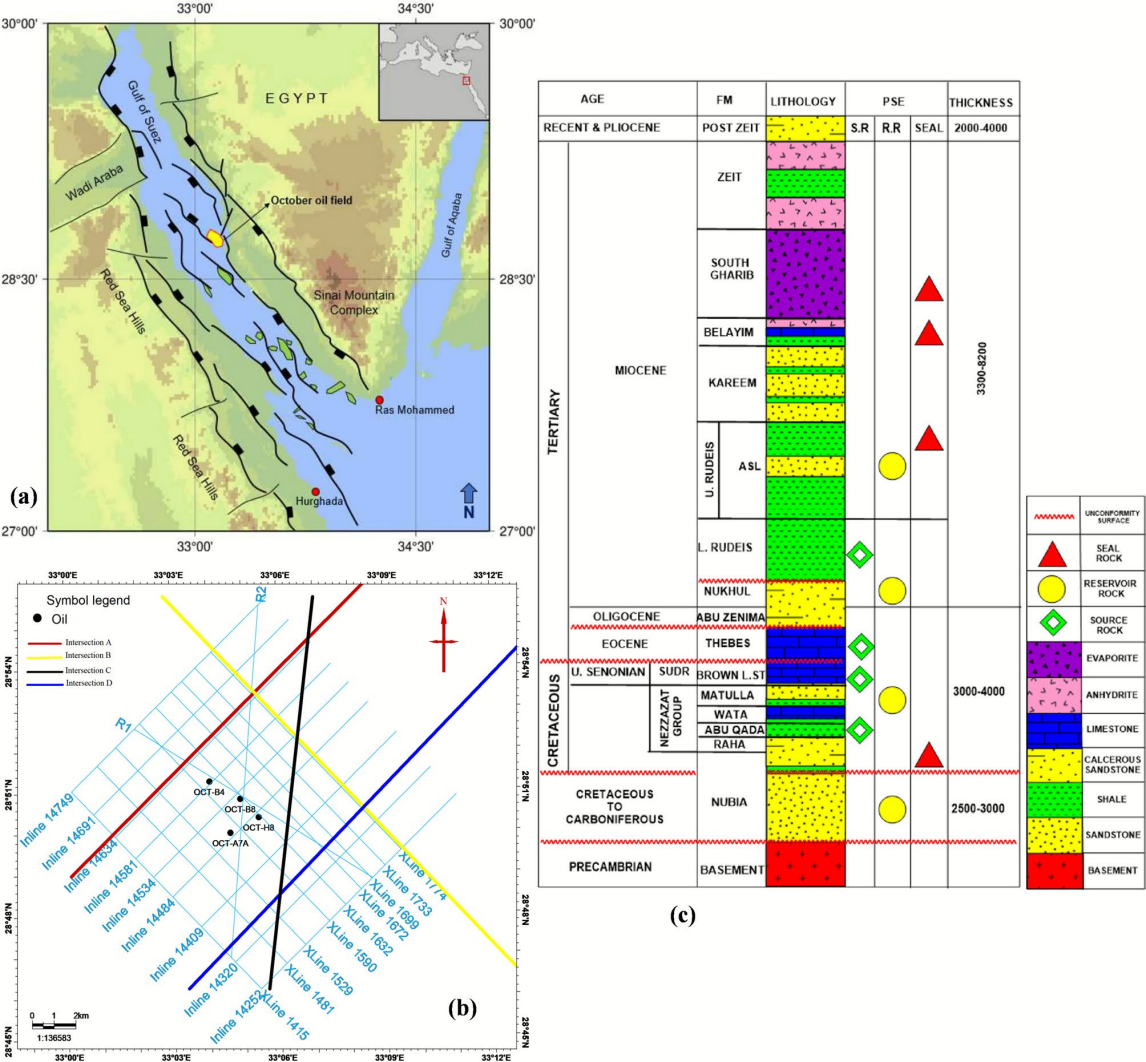
(**a**) Location of the October Field (yellow) within the structural framework of the Gulf of Suez Rift Basin (GSRB), showing major hydrocarbon fields (green) and regional faults. (**b**) Data distribution map illustrating the 2D seismic lines (black) and well locations (red) used in this integrated study. (**c**) Generalized tectono-stratigraphic column for the October Field, highlighting the pre-rift reservoir intervals (Nubia, Matulla) and key elements of the petroleum system.

The Gulf of Suez Rift Basin (GSRB) remains a globally significant hydrocarbon province, contributing substantially to Egypt’s petroleum production (e.g.,^1–4^). Within this mature basin, the October Oil Field (Fig. 1a) stands as a pivotal asset, discovered in 1977 and characterized by a complex interplay of extensional tectonics, diverse reservoir intervals, and intricate charge histories^5^. Decades of production have established its economic importance, yet significant uncertainties persist regarding compartmentalization, untapped resources across multiple pay zones, reservoir performance under secondary recovery, and the precise interplay of structure, stratigraphy, and diagenesis on fluid flow. A comprehensive, multi-disciplinary evaluation integrating seismic, well log, and core data is therefore essential to unlock remaining potential and guide future development strategies. Figure 1b shows seismic data and well location in the study area.

Structurally, the field is dominated by tilted fault blocks formed during the Late Oligocene–Miocene rifting phase. Recent three-dimensional structural modelling of structurally complex hydrocarbon reservoirs in the October Oil Field (e.g.,^6,7^) has significantly advanced our understanding of fault geometry and compartmentalization. However, translating this structural framework into accurate reservoir property distribution and connectivity remains challenging, particularly for pre-Miocene intervals like the pre-Cenomanian Nubian Sandstone ( e.g.,^7–10^). These deep reservoirs exhibit complex porosity–permeability relationships influenced by diagenesis and require detailed petrophysical evaluation and parameters relations investigation (e.g.,^3,11–17^).

The primary Miocene producing intervals include the Asl Member and Hawara Formation sandstones. Stratigraphic and petrophysical assessment of the ASL sandstone reservoir^12^ and evaluation of the ASL and Hawara formations using seismic- and log-derived properties (e.g.,^18,19^) have highlighted significant heterogeneity, lateral facies variations, and variable reservoir quality controlled by depositional environment and diagenetic overprints. Understanding this heterogeneity is critical not only for initial resource assessment but also for managing the effect of depletion and fluid injection in the Mesozoic and Paleozoic sandstone reservoirs (e.g.,^20–23^) and optimizing strategies like the environmentally friendly and economic waterflood system proposed for the field (e.g.,^20^). Production-induced stress changes and fluid-rock interactions pose risks to reservoir stability and sweep efficiency, necessitating integrated dynamic-static models.

Hydrocarbon charge is sourced primarily from the organic-rich Upper Cretaceous sequences, particularly during the Cenomanian/Turonian oceanic anoxic event 2 (OAE2) (e.g.,^20,24^). Detailed studies on hydrocarbon potentiality, burial history, and thermal evolution for some source rocks (e.g.,^16,20^) and hydrocarbon generation and charging (e.g.,^25–27^) have established the presence of effective source kitchens and variable thermal maturity across the field’s sub-blocks. However, migration pathways, charge timing relative to trap formation, and the potential for late-stage charge or re-migration into undrained compartments require further constraint through integrated basin modelling and geochemical fingerprinting.

While these individual studies provide valuable insights into specific aspects of the October Field, a critical gap exists in the holistic integration of structural architecture, reservoir characterization (spanning both Miocene and pre-Miocene units), source rock potential, charge history, and production dynamics. Previous efforts often focus on isolated datasets (e.g., seismic structure without core-calibrated petrophysics, or source rock geochemistry without robust migration modelling) or specific reservoir intervals. This fragmented approach limits the ability to accurately quantify remaining hydrocarbon potential and identify optimal redevelopment targets. This integrated approach provides a unified framework to reduce subsurface uncertainty. Furthermore, this study presents the first application of a fully integrated seismic-log-core workflow at this scale in the October Field to specifically resolve pre-rift compartmentalization and define predictive, HFU-based rock typing models.

Furthermore, the facies architecture and reservoir heterogeneity of these pre-rift units (Matulla and Nubia) must be understood within the broader tectono-stratigraphic framework of the Upper Cretaceous in the Gulf of Suez, which was characterized by the interplay of eustatic sea-level changes and syn-rift tectonic subsidence^28^. This framework explains the repeated shallow-marine clastic influxes and the localized development of reservoir quality observed in the October Field.

This study aims to bridge this gap by conducting a comprehensive, multi-scale evaluation of the October Field’s pre-rift reservoirs (Matulla and Nubia formations). We employ a synergistic analysis of 2D seismic, well log, and core data to:

1. Refine the structural model and define trap potential.
2. Perform core-calibrated petrophysical analysis to quantify reservoir properties.
3. Develop robust 3D structural-reservoir models to assess compartmentalization.
4. Implement a rigorous HFU analysis to define rock types with predictive permeability transforms.

This integrated workflow provides a unified framework to reduce subsurface uncertainty, optimize recovery from existing reservoirs, and delineate new exploration targets within this mature but highly prospective field. Our approach advances beyond previous work by quantitatively linking the seismic-scale structural framework to the pore-scale geometric attributes that ultimately govern fluid flow, offering a transferable methodology for re-evaluating mature rift basins globally.

## Geologic setting

The October Field is situated in the central GSRB, a NNW-SSE trending extensional basin formed during Afro-Arabian plate separation in the Late Oligocene (∼28–23 Ma)^1^. This mature hydrocarbon province exhibits a classic rift asymmetry, with the October Field residing on the gently dipping western margin, characterized by a series of NW–SE oriented, basement-involved tilted fault blocks^2,29^.

### Tectono-stratigraphic evolution

The basin’s stratigraphy reflects three distinct phases^30–32^, (Fig. 1c)^3,14,33^:

1. Pre-rift (> 28 Ma): Comprises Neoproterozoic basement overlain by thick Cambrian to Eocene passive margin sequences. The primary pre-rift reservoir is the Pre-Cenomanian Nubian Sandstone (Lower Cretaceous) a continental to shallow-marine quartz arenite deposited in a fluvial-deltaic system exhibiting variable porosity (12–25%) and permeability (10–500 mD) due to quartz cementation and kaolinite occlusion^34^.

The Upper Cretaceous succession, including the Matulla and Wata formations, was deposited during a period of regional subsidence and marine transgression preceding the main Gulf of Suez rifting. This interval records the interplay of eustatic sea-level changes and localized tectonic subsidence on the evolving rift margin^35^. The Matulla Formation in the October Field represents a complex mix of shallow marine, shoreline, and fluvial-deltaic clastics, correlative with the Wata Formation in other parts of the basin. This tectono-stratigraphic setting explains the pronounced heterogeneity and lateral facies variability observed in these reservoirs, as documented in studies of analogous intervals^36^. The deposition of these sandstones was influenced by the creation of accommodation space through early syn-rift subsidence, which controlled sediment supply and distribution, leading to the compartmentalized reservoir architecture that is a focus of this study.

2. Syn-rift (28–14 Ma): Dominated by Miocene clastic-carbonate cycles deposited in evolving half-grabens. Key reservoirs include:

- Asl member (Upper Rudeis Fm.): Braided-delta to shoreface sandstones, averaging 20% porosity but with strong permeability anisotropy^37–39^.
- Hawara formation*:* Shallow-marine carbonates with fracture-enhanced permeability^14,40^. Source rocks are concentrated in the Upper Rudeis and Kareem formations (Type II kerogen, TOC: 2–5%) and the deeper Cenomanian/Turonian (OAE2) Brown Limestone (Type II-S, TOC: 4–8%), deposited during oceanic anoxic event 2^16,20,25,26^.

3. Post-rift (< 14 Ma): Evaporites (South Gharib Fm.) providing regional seals, overlain by Pliocene-Recent marls^1^.

Three-dimensional structural modelling reveals a complex array of NNW-striking, domino-style rotated blocks bounded by high-angle (45°–65°) normal faults with throws exceeding 300 m^37,41^. Fault reactivation during the Late Miocene (post-10 Ma) created secondary synthetic/antithetic faults, compartmentalizing reservoirs into isolated pressure cells. This architecture critically controls:

- Hydrocarbon migration pathways from Cretaceous kitchens
- Trap integrity via shale smear potential (SGR > 0.25)
- Reservoir connectivity, particularly in the Nubian Sandstone.

### Petroleum system elements

- Source: Thermal maturation modelling indicates peak oil generation from the Brown Limestone (OAE2) occurred during the Late Miocene (8–5 Ma), synchronous with trap formation^16,20,24,25^.
- Reservoirs: Miocene sandstones (Asl, Hawara) exhibit porosity reduction from 30% (depositional) to 15–22% due to quartz overgrowths, while Nubian Sandstone quality is controlled by early chlorite rims inhibiting cementation^18^.
- Seals: Evaporites (South Gharib) and marine shales (Kareem) provide top seals, with intraformational shales causing vertical compartmentalization^1^

### Production challenges

Field maturity has led to declining pressures (∼35% depletion in Nubia reservoirs), inducing stress-sensitive permeability reduction and wellbore instability during fluid injection for pressure maintenance^20^. Understanding diagenetic heterogeneity and fault seal integrity is thus critical for optimizing waterflood systems and targeting undrained compartments^20,37^.

## Materials and methods

This study employs an integrated, multi-scale workflow to characterize pre-rift reservoirs in the October Field (Fig. 1a). The methodology bridges seismic-scale structural architecture, well-log-scale petrophysical properties, and core-scale pore system analysis, synthesizing data from 20 2D seismic lines, 4 wells, and 302 core plugs.

### Seismic interpretation

Seismic interpretation focused on delineating the structural framework and key reservoir horizons (Matulla and Nubia formations) across the October Field using 20 depth-migrated 2D seismic lines, covering an area of about 150 Km^2^. Depth-domain seismic lines (SEG-Y format) were loaded into Petrel™ 2017 and data quality was assessed. Both Matulla and Nubia reservoirs were picked up and the faults affecting them were identified. Depth-structure contour maps were created on top of Matulla and Nubia formations. All seismic interpretation, visualization, and map generation were conducted using the Petrel™ 2017 E&P software platform (SLB).

### Well log analysis

This study employs a curated dataset extracted from four strategically positioned boreholes within the target basin. Each well provides a comprehensive suite of high-resolution geophysical logs—gamma-ray (GR), resistivity, neutron, density, and sonic—archived in industry-standard LAS and ASCII formats. The initial phase involved comprehensive log quality control (QC) and environmental corrections to ensure data integrity. These datasets facilitated an integrated analytical protocol, merging qualitative lithological interpretation with quantitative petrophysical assessment. Shale volume (Vsh) was calculated from the gamma-ray log using a normalized linear relationship. Total porosity (Φt) was derived primarily from the density log, calibrated to core data. Effective porosity (Φe) was then calculated by correcting Φt for the shale volume. Water saturation (Sw) was determined using the Archie equation, with parameters (a, m, n) selected based on core measurements and regional experience, and formation water resistivity (Rw) determined from Pickett plots. The methodology enabled rigorous delineation and characterization of hydrocarbon-bearing intervals, with focused analysis on the stratigraphically heterogeneous Matulla and Nubia formations.

Formation evaluation was anchored by a suite of petrophysical parameters: shale volume (Vsh), total and effective porosity (Φt, Φe), water saturation (Sw), hydrocarbon saturation (Sh), and net-to-gross pay ratio (N/G). These metrics quantitatively characterize clay content, pore-space geometry, and pore-fluid distribution^42–45^.

### Static reservoir modeling

This study implemented integrated 3D structural, facies, and petrophysical property modeling as the core methodology to achieve its objectives. Seismic and petrophysical data integration provided critical constraints for facies and property modeling by delineating lithotype distributions and quantifying reservoir heterogeneity. Static reservoir modeling—a definitive component of reservoir characterization—was employed to characterize reservoir architecture and spatial variability in key parameters^13,14,40,42,44^. The workflow commenced with quantitative facies modeling, utilizing well-derived lithology logs (e.g., gamma-ray, neutron-density) from all wells penetrating the Matulla and Nubia reservoirs as conditioning data. Geostatistical methods (sequential indicator simulation) rigorously honored vertical and lateral facies trends identified in core and log datasets. Subsequently, petrophysical properties were modeled through co-kriging^4,7,13,46^, ensuring spatial correlation with facies architecture.

### Core analysis and hydraulic flow unit (HFU) characterization

Conventional core analysis (CCAL) was conducted on 320 core plug samples representing the Nubia Formation in well OCT-B8. All core plugs were cleaned, dried, and extracted using standard solvents to remove residual hydrocarbons and salts prior to measurement. Subsequent hydraulic flow unit analysis was performed on a subset of 208 samples that met reservoir-grade quality criteria; the remaining samples were excluded as they represented non-reservoir lithologies (e.g., shales) or yielded failed measurements. The analysis provided fundamental petrophysical properties: total porosity measured by helium porosimetry, absolute permeability (k) to nitrogen determined by steady-state gas flow, and grain density (ρ_g) obtained via helium pycnometry.

Grain density (ρ_g), defined as the mass per unit volume of the solid rock matrix excluding pore space, is a fundamental petrophysical property critical for accurate porosity calculation and mineralogy interpretation^47–50^. Due to inherent mineralogical heterogeneity within samples, grain density values often exhibit a distribution rather than a single value. Histogram analysis of bulk grain density measurements across representative core plugs is the established method to identify the dominant mineral composition^51^.

Correlating core-derived porosity with log-derived porosity is essential for calibrating petrophysical models and validating log responses across a reservoir interval. Core porosity, measured under controlled laboratory conditions, is considered the ground truth^52,53^.

While “rock quality” generally describes a reservoir’s storage and flow capacity (e.g., high porosity and permeability), “rock typing” is a systematic process to classify rocks into distinct units. Rock typing (RT) is the systematic classification of reservoir rocks into distinct units (“Rock Types” or RTs) based on their intrinsic pore geometry, which governs fluid storage (porosity) and flow capacity (permeability, k) behavior. Unlike lithofacies based primarily on depositional texture and composition, hydraulic rock types aim to group rocks with similar fluid flow characteristics resulting from both depositional and diagenetic processes (e.g., cementation, dissolution)^43,54,55^.

Multiple methodologies exist for effective rock typing. Geological approaches, such as the Lucia classification, utilize reservoir depositional and diagenetic characteristics. Petrophysical techniques predominantly leverage core-derived properties, particularly porosity and permeability. Additionally, empirical and theoretical frameworks have been developed to systematize petrophysical rock typing (PRT). Rocks assigned to a specific rock type share defining static properties (e.g., pore geometry, mineralogy) and dynamic properties (e.g., capillary pressure, relative permeability), which collectively govern fluid flow behavior. These distinct property sets are formally categorized as Petrophysical Static Rock Types (PSRT) and Petrophysical Dynamic Rock Types (PDRT)^43,56^.

The Flow Zone Indicator (FZI) provides a quantitative framework linking pore-scale geometric attributes (e.g., pore and throat size distribution, aspect ratio, tortuosity) to macroscopically measurable reservoir properties, specifically porosity and permeability. It is derived from a modified Kozeny-Carman (KC) equation:

$$k=\upphi \frac{{r}_{ms}2}{{F}_{s}t} (1)$$

where r_mh_ is the mean hydraulic radius, F_s_ is the pore shape factor, and t is the hydraulic tortuosity, the ratio of the actual length to the straight length (L_a_/L). FZI enables the prediction of pore-scale characteristics from CCAL data^57^. Its calculation requires first determining the Reservoir Quality Index (RQI) and Normalized Porosity (ϕ_z_)^58^:

2 $$\text{RQI}=0.0314\sqrt{K/\upphi }$$

3 $$\upphi_{{\text{z}}} = \upphi / \, ({1} - \upphi )$$

4 $${\text{FZI }} = {\text{ RQI}}/\upphi_{z}$$

Rocks with similar FZI values possess comparable pore geometry and constitute distinct HFUs. Significant research efforts have focused on refining the FZI formulation, aiming to either enhance its physical basis or reduce parameterization complexity^59–62^. This advancement is critical as FZI-based rock typing provides an essential methodology for predicting reservoir properties (notably permeability) in uncored intervals, mitigating reliance on expensive coring programs.

Complementing pore-geometry methods like FZI, the Stratigraphic Modified Lorenz (SML) plot offers a robust technique for delineating reservoir sequences into HFUs based on their dynamic flow-storage characteristics. The SML method involves the cumulative summation of flow capacity (k * h) versus storage capacity (ϕ * h) along a depth sequence. Distinct HFUs are identified as linear segments within this plot, with the slope of each segment (d(Σk h)/d(Σϕ h)) representing the unit’s effective permeability-to-porosity ratio (k/ϕ) and thus its flow efficiency. Steeper slopes correspond to higher hydraulic conductivity, enabling the classification of intervals as non-conductive (e.g., barriers), conductive, or super-conductive zones^54,63^. The efficacy and widespread applicability of the SML method for HFU identification have been extensively validated across diverse reservoir types^34,64–68^.

## Results

### Seismic interpretation

**Fig. 2:**
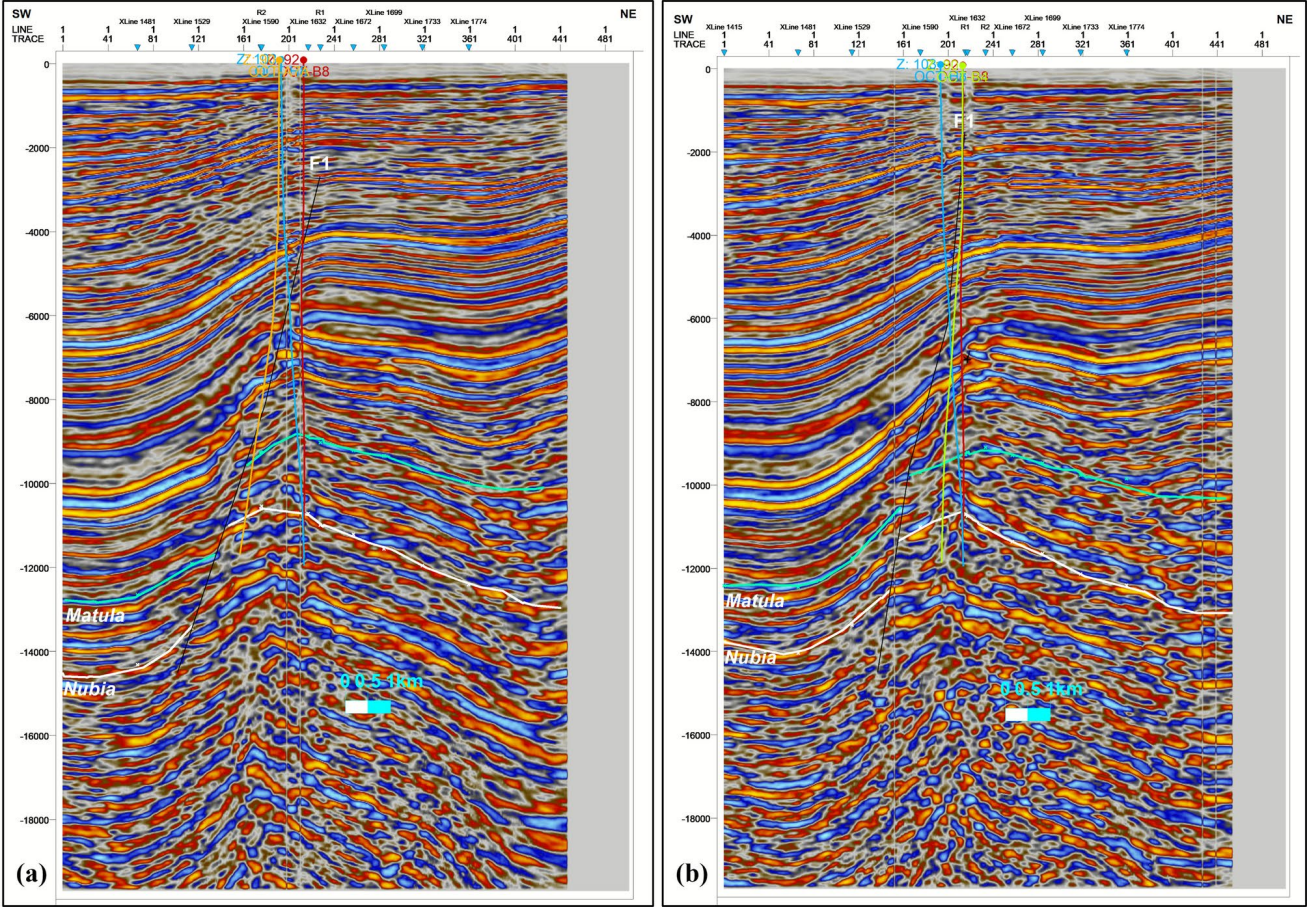
Interpreted seismic sections showing key horizons and the fault systems compartmentalizing the Matulla and Nubia reservoirs.

**Fig. 3:**
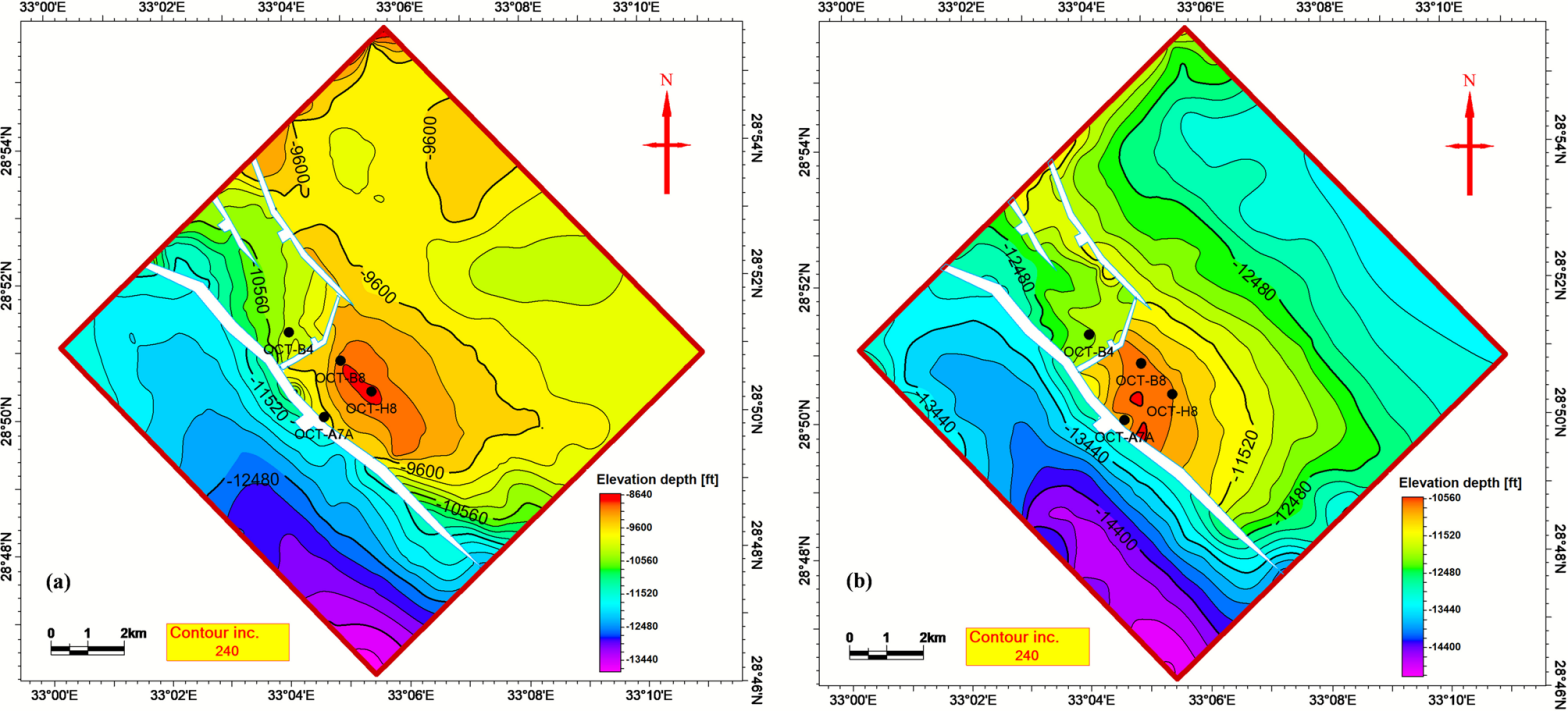
Depth-structure maps revealing fault-bounded structural traps at the top of the (**a**) Matulla and (**b**) Nubia formations.

Seismic interpretation indicates that the key Matulla and Nubia formation tops within the study area are offset by two distinct sets of normal faults. Figure 2 presents representative interpreted seismic sections, illustrating the mapped Matulla and Nubia horizons and the associated fault networks affecting them. As the seismic data is depth-migrated, depth structure contour maps for both the Matulla and Nubia formation tops (Fig. 3a, b, respectively) were generated directly from the interpreted horizons, eliminating the need for time-depth conversion. These contour maps (Fig. 3) reveal the presence of four dominant normal faults. Three of these faults exhibit a NW–SE orientation, consistent with the dominant structural trend of the Gulf of Suez rift system. The fourth fault displays a NE-SW orientation, aligning with the structural trend characteristic of the Gulf of Aqaba.

### Petrophysical evaluation

#### Matulla Formation

**Fig. 4:**
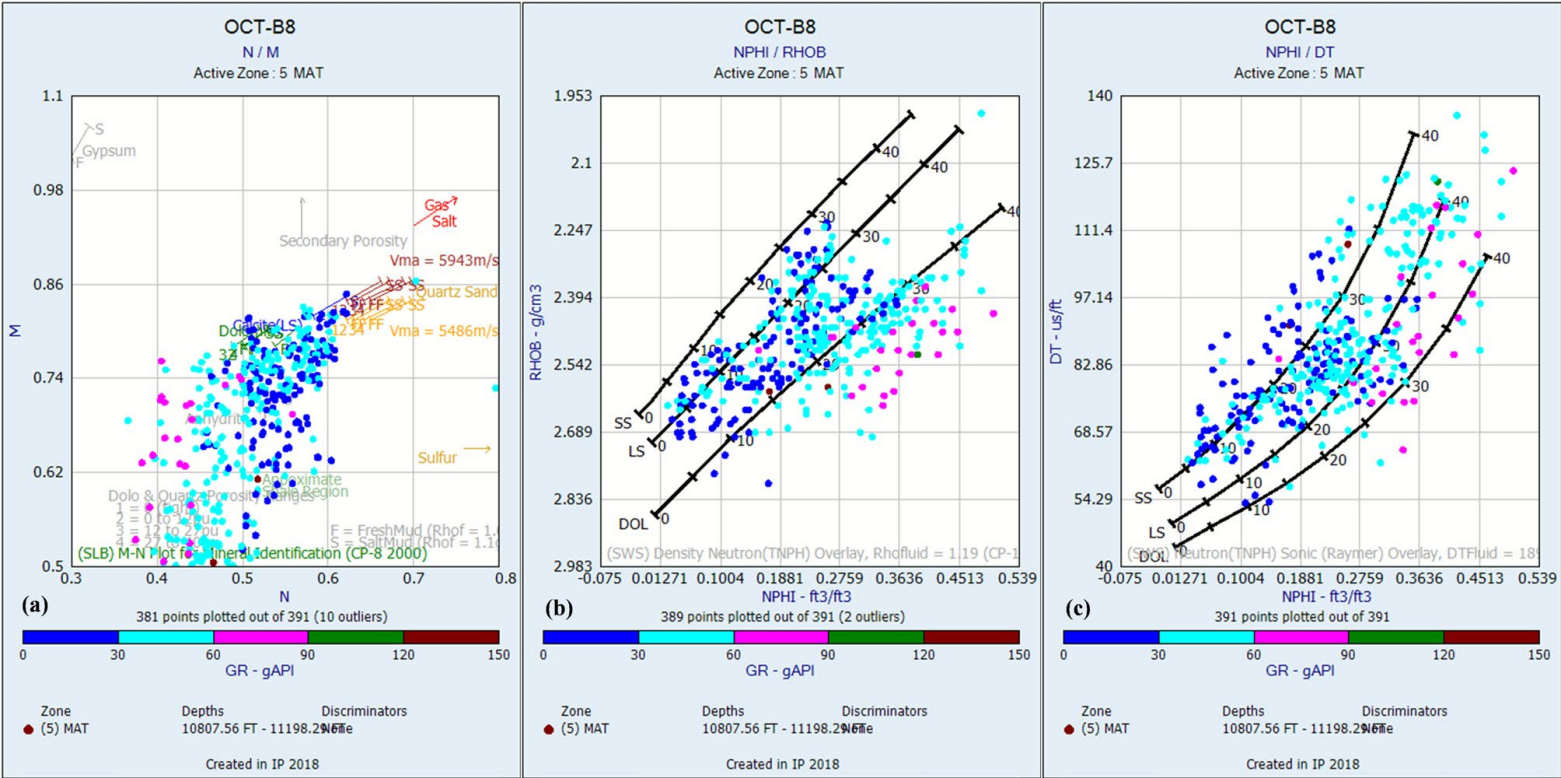
Lithofacies discrimination for the Matulla Formation using (**a**) M–N, (**b**) Neutron-Density, and (**c**) Neutron-Sonic cross-plots.

Standard petrophysical cross plots (MN, Neutron-Density, Neutron-Sonic; Fig. 4a,b,c, respectively) indicate that the Matulla Formation is dominated by sandstone and shale lithologies. The Matulla Formation exhibits variable reservoir characteristics across the evaluated wells. Net Pay thickness ranges from 150 ft (OCT-B8) to 262.5 ft (OCT-H8), indicating moderate reservoir development. Shale Volume (Vsh) is notably higher than in the Nubia, averaging ~ 16% (excluding OCT-B4, where Matulla Formation does not exist), suggesting a more significant clay content influencing reservoir quality. Despite this, the Matulla demonstrates favorable Effective Porosity, averaging ~ 20% across wells OCT-A7A, OCT-H8, and OCT-B8, with a maximum of 23% in OCT-A7A. The determination of formation water resistivity (Rw), a critical parameter for subsequent water saturation calculations, was achieved through rigorous analysis of Pickett plots (resistivity-porosity cross plots)^69^. Robust interpretation of these plots yielded distinct Rw values for the evaluated formations: a value of 0.0227 Ω·m was determined for the Matulla Formation (Fig. 6a). Hydrocarbon potential is indicated by consistently high Oil Saturation values, ranging from 71 to 76% in the wells with data (Table 1). Petrophysical data for the Matulla Formation is absent in well OCT-B4. This is because the formation itself is absent in this well.

**Table 1:** The average petrophysical parameters estimated for Matulla and Nubia formation in the study area.

Formation	Well name	Net pay (ft)	Shale volume (%)	Effective porosity (%)	Oil saturation (%)
Matulla	OCT-A7A	232	14	23	71
	OCT-B4	–	–	–	–
	OCT-H8	262.5	19	18	76
	OCT-B8	150	14	18	76
Nubia	OCT-A7A	395	5	19	68
	OCT-B4	484	6	19	85
	OCT-H8	314	2	18	65
	OCT-B8	310	8	16	69

#### Nubia Formation

**Fig. 5:**
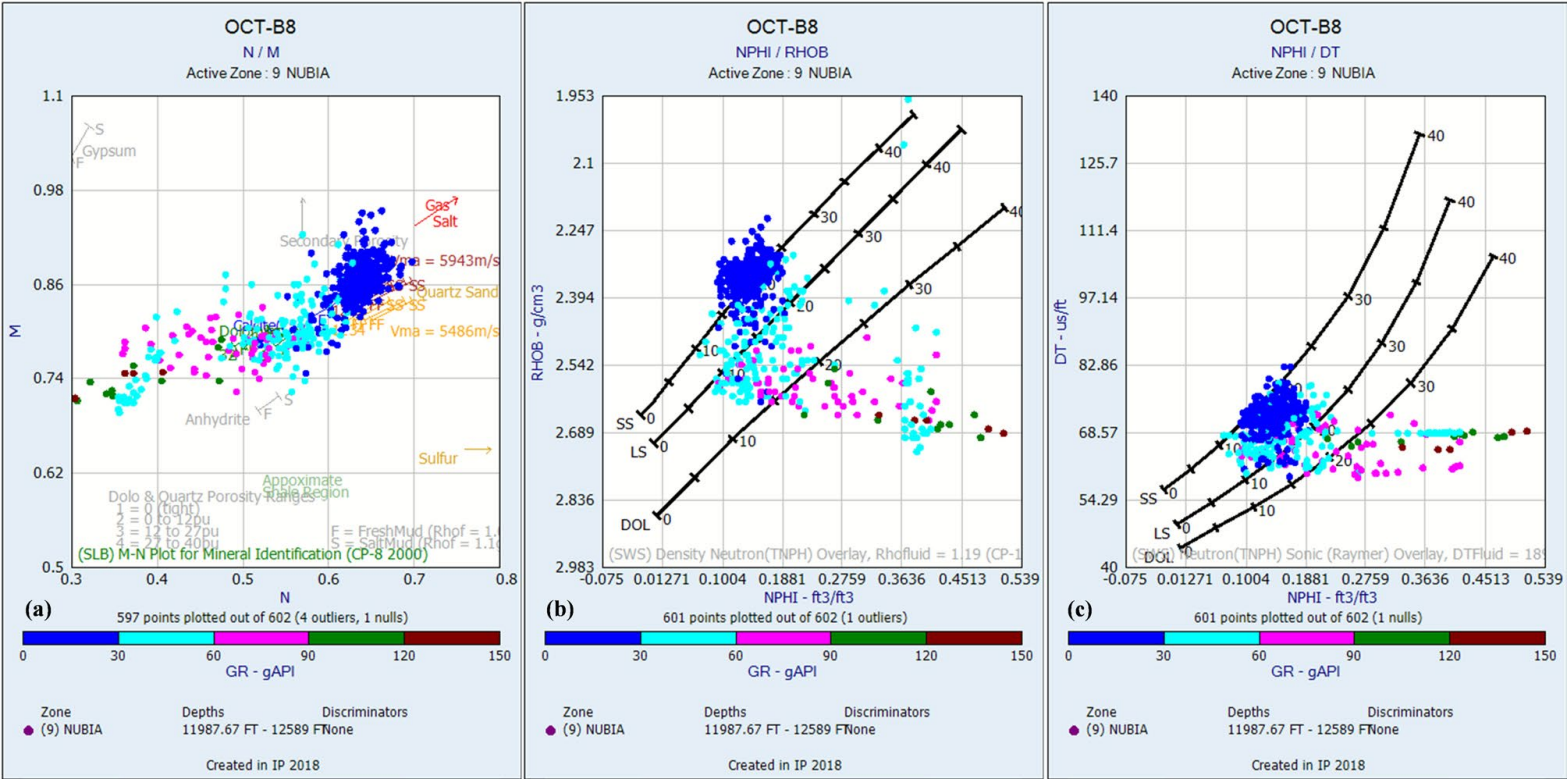
Lithofacies discrimination for the Nubia Formation using (**a**) M–N, (**b**) Neutron-Density, and (**c**) Neutron-Sonic cross-plots.

**Fig. 6:**
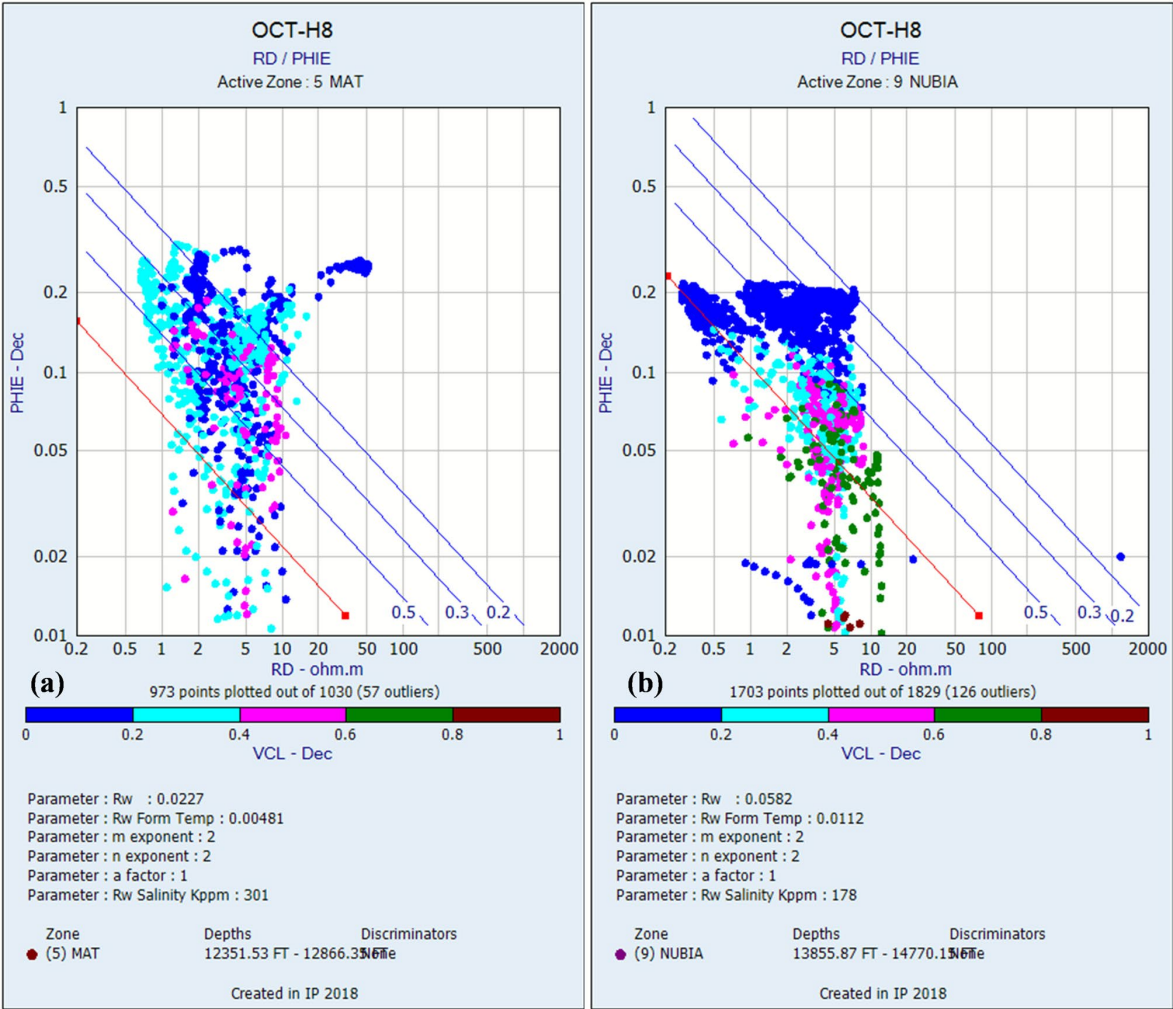
Pickett plots for the (**a**) Matulla and (**b**) Nubia formations, used to determine formation water resistivity (Rw).

Standard petrophysical cross plots (MN, Neutron-Density, Neutron-Sonic; Fig. 5a,b,c, respectively) indicate that the Nubia Formation is dominated by sandstone and shale lithologies, with minor carbonate intervals. Nubia Formation displays superior reservoir properties overall compared to the Matulla. Net Pay Thickness is significantly greater and more consistent, ranging from 310 ft (OCT-B8) to 484 ft (OCT-B4) (Table 1), highlighting its substantial reservoir volume potential. Critically, Shale Volume (Vsh) is markedly lower, averaging ~ 5% across all four wells, indicating cleaner, higher-quality sandstones. Effective Porosity is good, averaging ~ 18%, though slightly lower than the best Matulla intervals. Robust interpretation of Pickett plots yielded distinct Rw values for Nubia Formation of 0.0582 Ω·m (Fig. 6b). These quantitatively derived Rw estimates, reflecting differing formation water salinities, provide the essential foundation for accurate petrophysical evaluation of hydrocarbon-bearing zones within each reservoir interval. Oil Saturation is generally high (65–69%) in three wells and exceptionally high at 85% in well OCT-B4 (Table 1), identifying a potential local sweet spot. The combination of significant thickness, low shale content, good porosity, and high oil saturation establishes the Nubia as the more robust hydrocarbon-bearing unit within the study area.

**Fig. 7:**
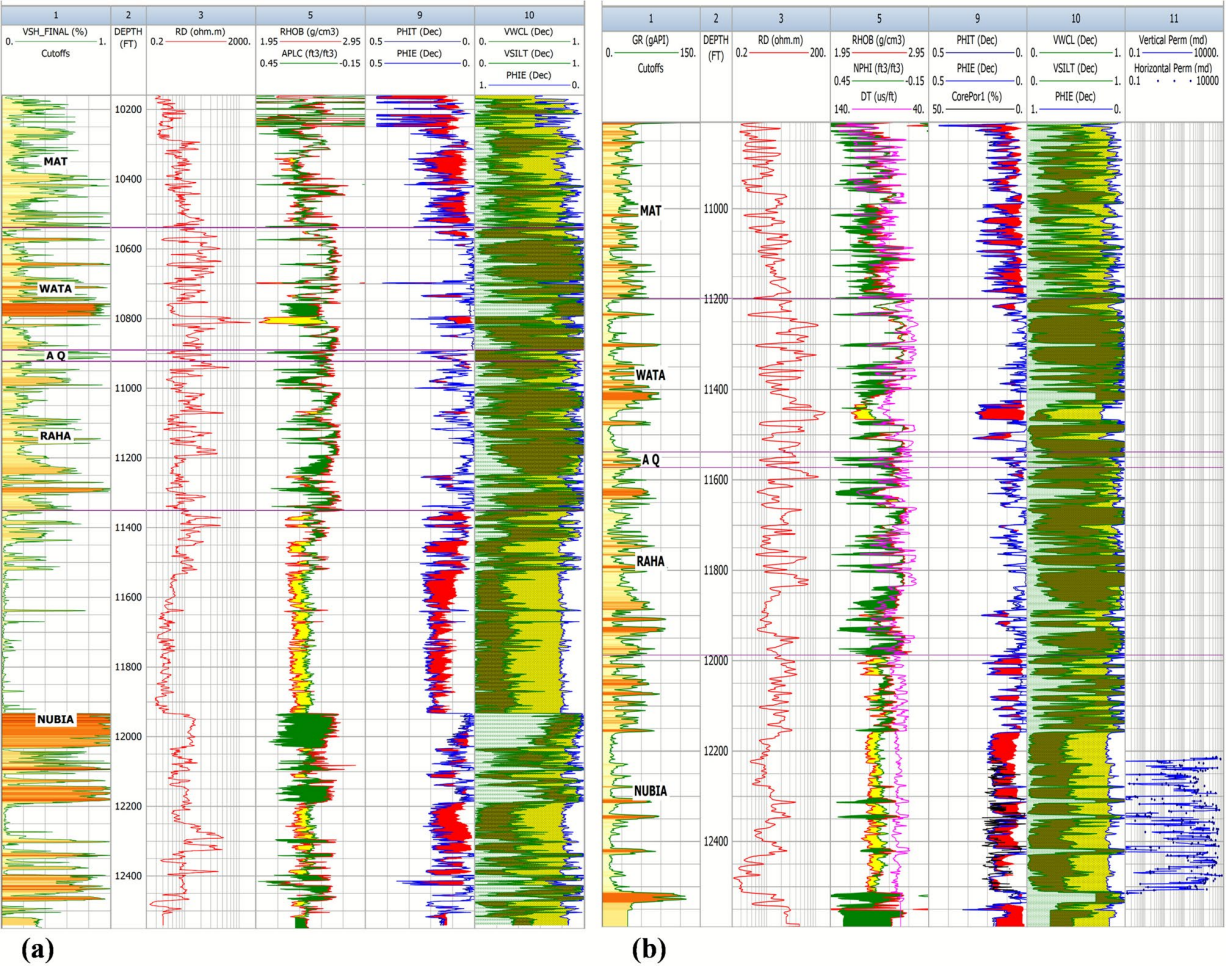
Lithosaturation panels for two representative wells, illustrating the vertical distribution of petrophysical parameters.

In conclusion, petrophysical analysis confirms both the Matulla and Nubia formations as viable hydrocarbon reservoirs within the study area. However, the Nubia Formation demonstrates superior overall reservoir characteristics, particularly in terms of net thickness and reservoir rock quality (lower Vsh), making it the primary target for resource assessment and development focus. The localized high oil saturation in the Nubia (OCT-B4) warrants further investigation. The vertical distribution of the estimated petrophysical parameters is depicted in the lithosaturation panel of two wells in the study area (Fig. 7).

### Reservoir modeling

**Fig. 8:**
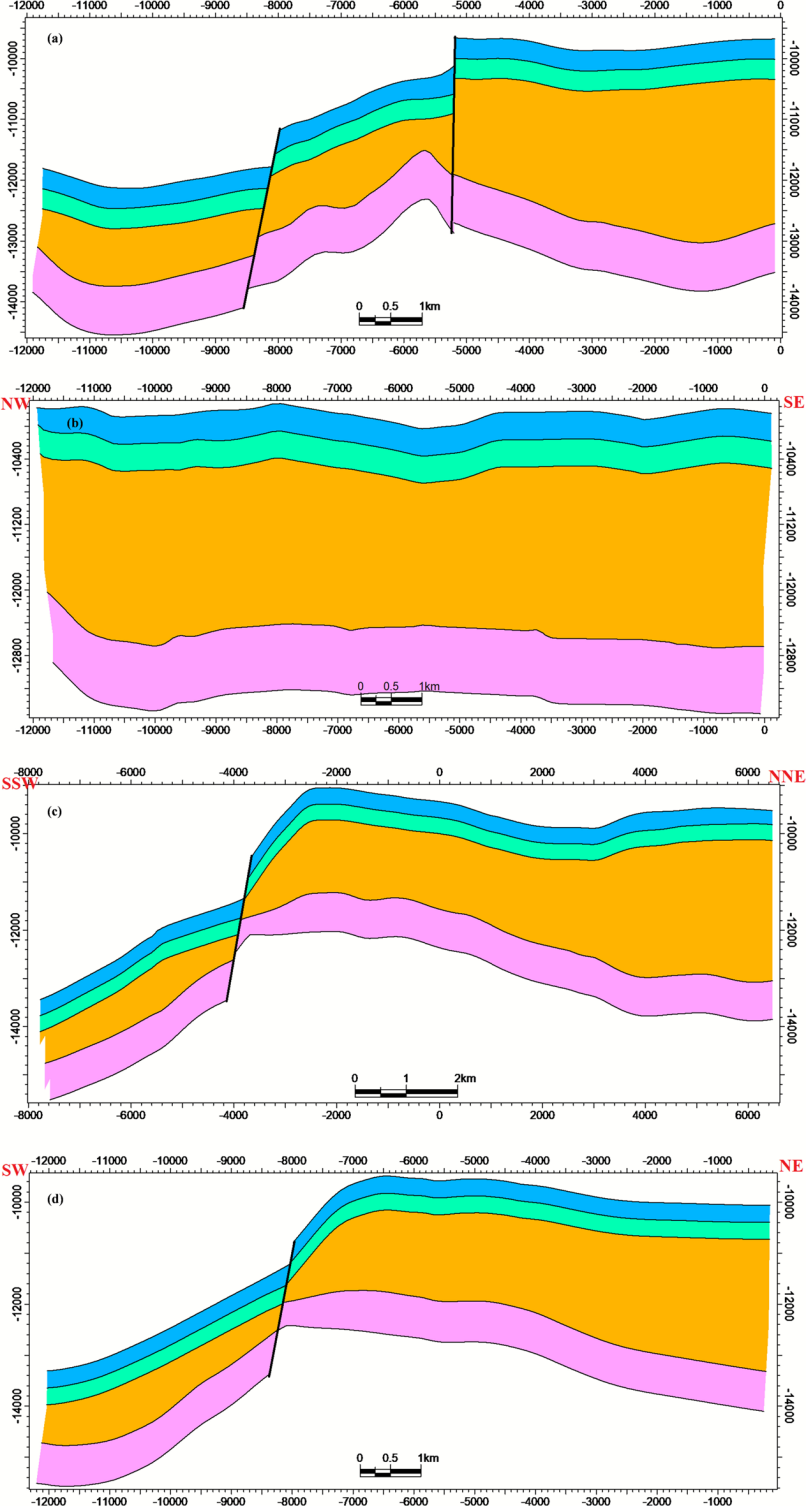
Structural model intersections highlighting significant lateral variation in fault-block geometry across the study area.

**Fig. 9:**
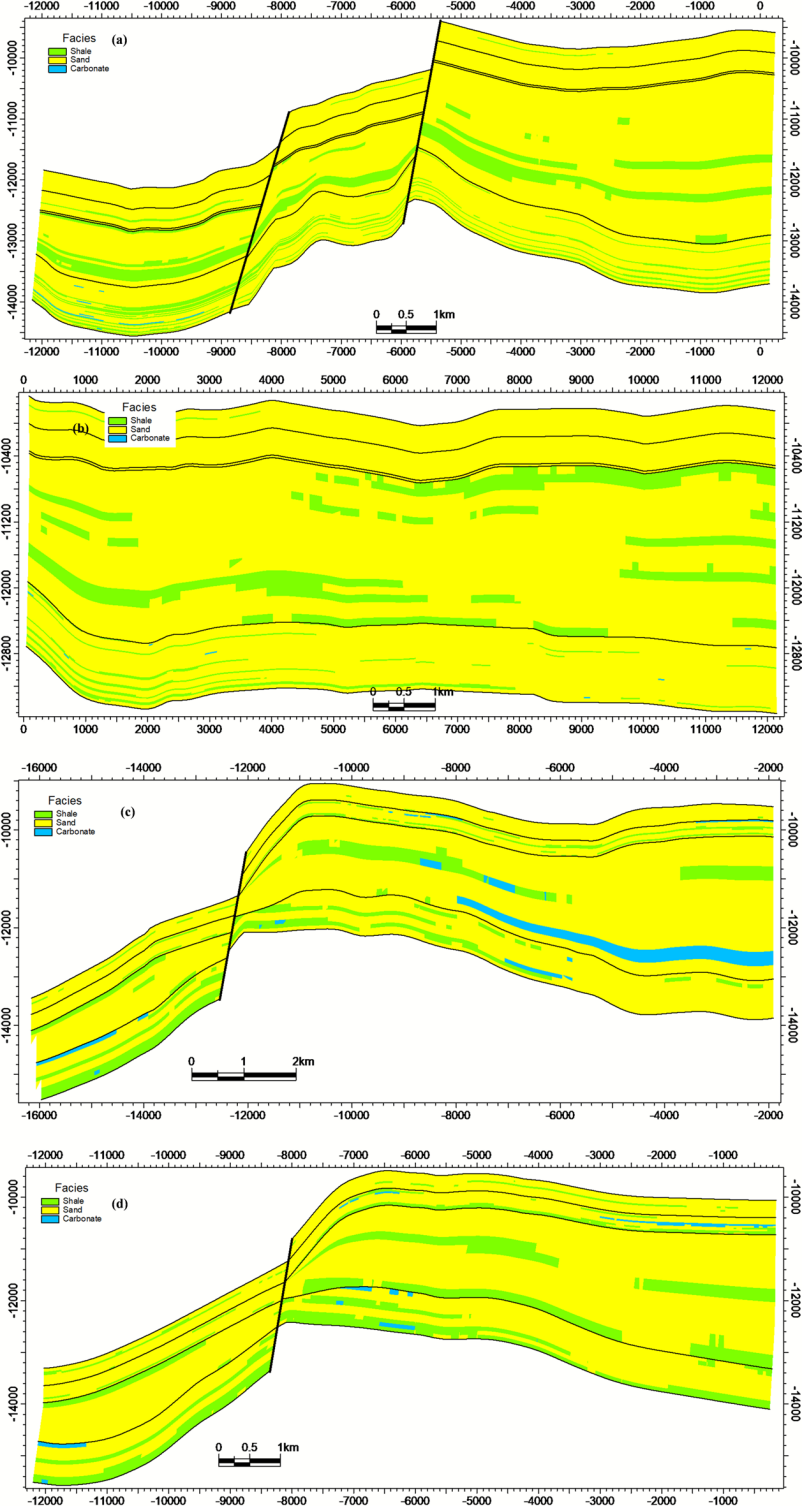
Facies model intersections showing the heterogeneous distribution of sandstone, shale, and limestone.

The structural framework defining the Matulla Formation top to Nubia Sandstone base is dominated by NW–SE oriented normal faults, generating a system of step faults. Cross-sections a-d (Fig. 8) delineate significant lateral variations in the structural geometry across the study area. Lithofacies distribution within the Matulla-Nubia interval comprises three primary components: (1) fluvial-deltaic sandstone, (2) lagoonal shale, and (3) minor limestone occurrences (< 5%). Figure 9a-d document pronounced spatial heterogeneity in facies proportions and architecture across the respective intersections.

**Fig. 10:**
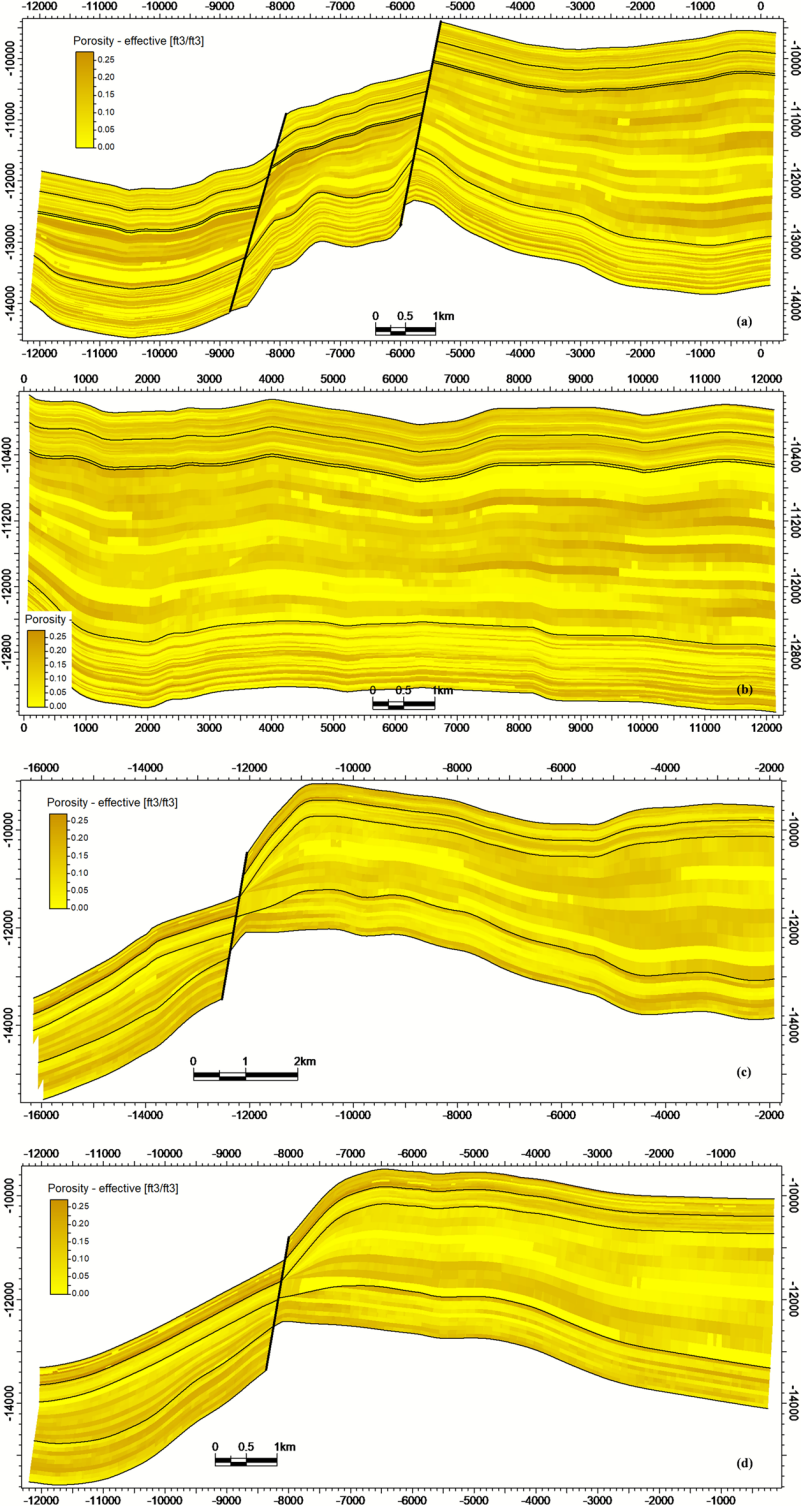
Porosity model intersections demonstrating facies-controlled porosity distribution, with highest values in Nubia sandstones.

Effective porosity ranges from 5 to 22%, with values strongly dictated by lithofacies. Sandstone intervals, particularly within the Nubia Formation, exhibit the highest porosity (15–22%), while shale and limestone facies range from 5 to 12%. The spatial distribution of effective porosity across intersections a-d is detailed in Fig. 10a-d.

**Fig. 11:**
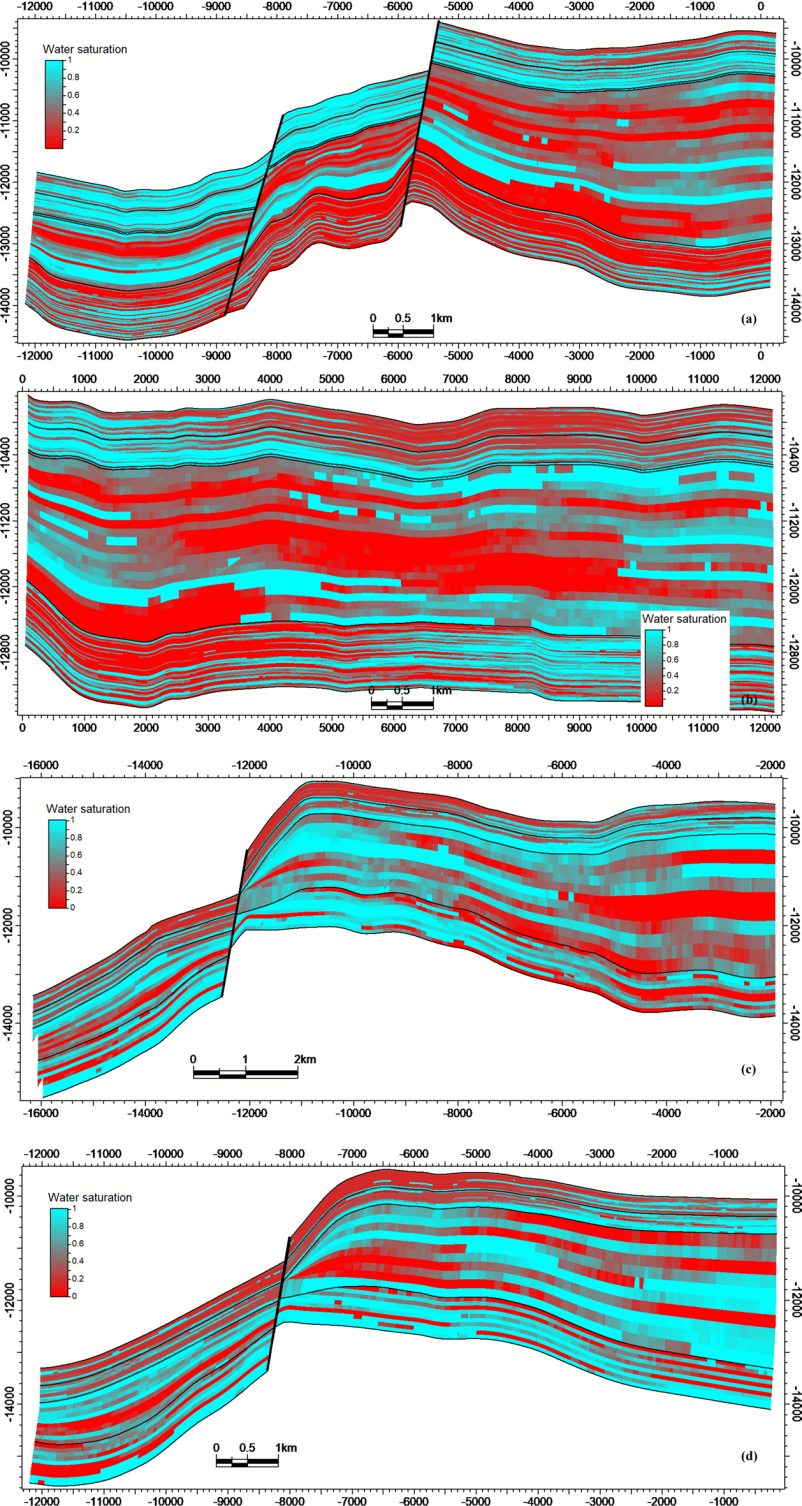
Hydrocarbon saturation (S ~ h ~) model intersections, showing S ~ h ~  > 80% concentrated in structurally elevated, high-porosity zones.

Hydrocarbon saturation (Sₕ) reaches maxima (70–85%) within high-porosity sandstone units contained by structural closures (Fig. 11a-d). Significant heterogeneity in Sₕ correlates directly with lithofacies architecture (Fig. 9) and structural compartmentalization (Fig. 8). Collectively, the integrated models establish that reservoir quality (Fig. 9, 10) and fluid distribution (Fig. 11) are predominantly controlled by structural segmentation (Fig. 8) and facies-dependent diagenetic processes. Optimal reservoir targets are preferentially localized within high-porosity, fault-bounded sandstone units of the Nubia Formation (intersections a-b), where Sₕ consistently exceeds 80%.

### Core analysis and rock typing

Laboratory-based core analysis provides fundamental petrophysical parameters and serves as the benchmark for validating log-derived interpretations. For the Nubia Formation, a comprehensive suite of core measurements was leveraged to achieve these objectives.

**Fig. 12:**
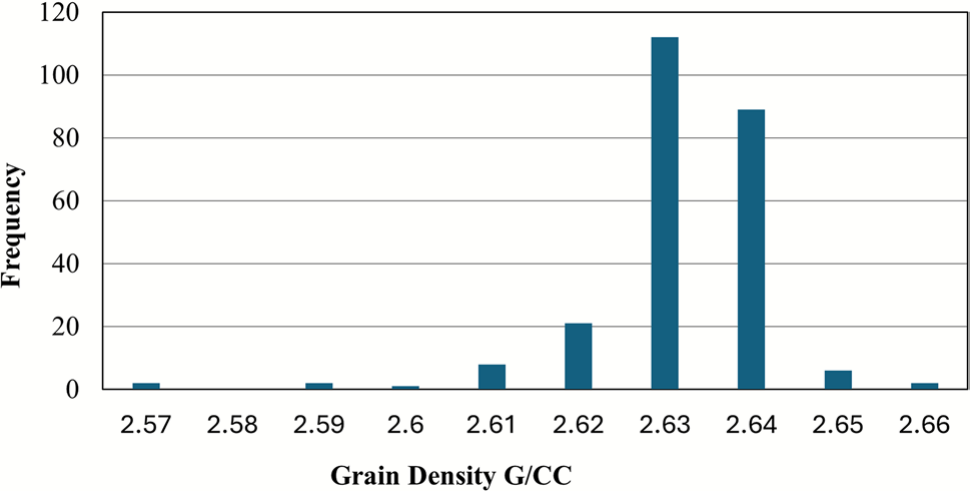
Grain density (ρ ~ g ~) distribution for the Nubia Formation, indicating a dominant quartz composition.

To establish a robust lithological reference for porosity calculations derived from density logs, grain density (ρg) was analyzed from core plug samples representing key intervals within the Nubia Formation. Grain density, representing the intrinsic density of the mineral matrix, is a critical parameter significantly influencing the accuracy of porosity calculations from density logs. A statistical distribution of grain density measurements was constructed, generating a histogram to characterize the lithological variation (Fig. 12). Analysis of this distribution revealed a distinct central tendency. The mean grain density for the Nubia Formation was determined to be 2.63 g/cm^3^, indicative of a relatively homogeneous quartz-dominated mineralogy. This empirically derived value was subsequently applied as the matrix density input parameter for all log-based density-porosity transforms within the Nubia Formation.

**Fig. 13:**
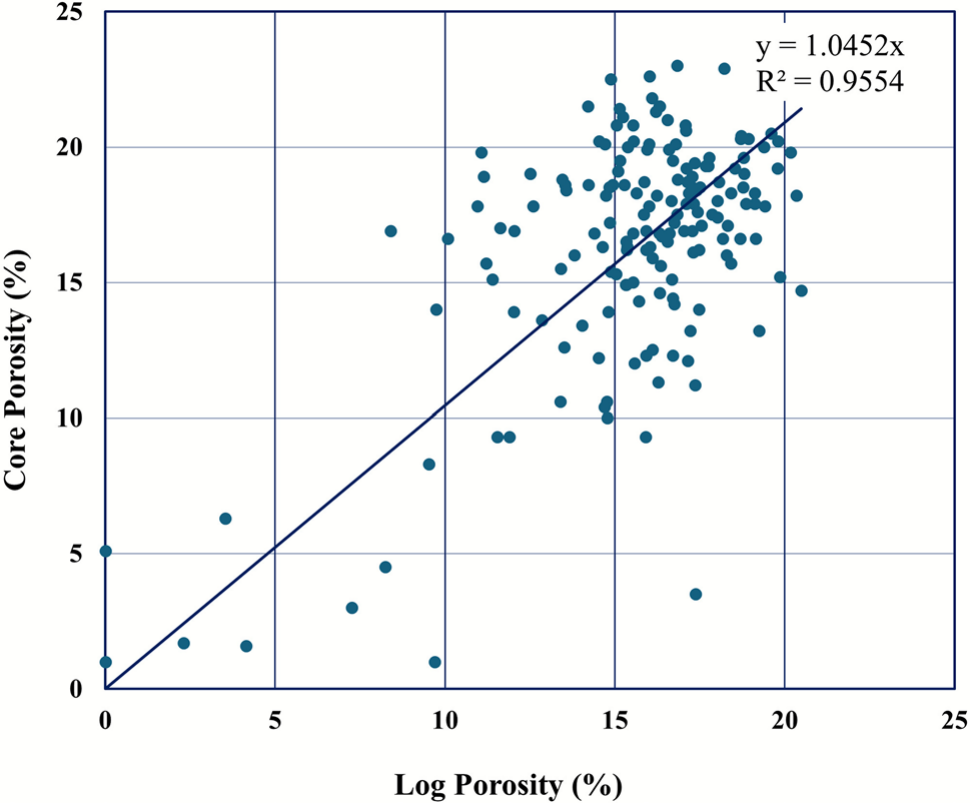
Core-log porosity cross-plot validating the petrophysical model with a high correlation coefficient (R^2^ = 0.955).

A critical step in ensuring the reliability of the petrophysical model was the rigorous integration and comparison of core-derived porosity with log-derived total porosity. A comparative analysis was performed across the cored well within the Nubia Formation to encompass natural lithological variability. Regression analysis of the paired core porosity and log porosity data points demonstrated an exceptionally strong linear relationship (Fig. 13). The coefficient of determination (R^2^) was 0.955. This high-fidelity agreement provides robust quantitative validation of the log-derived porosity calculations and underscores the accuracy and consistency of the log data acquisition, environmental corrections, and petrophysical processing methodology employed for the Nubia Formation.

The core-derived mean grain density establishes a lithologically constrained foundation for porosity determination. The exceptionally high correlation (R^2^ = 0.955) between core and log porosity provides compelling evidence for the reliability of the log-derived petrophysical parameters. This successful core-log integration significantly enhances confidence in the subsequent calculations of water saturation and hydrocarbon pore volume within the Nubia Formation reservoir model.

**Fig. 14:**
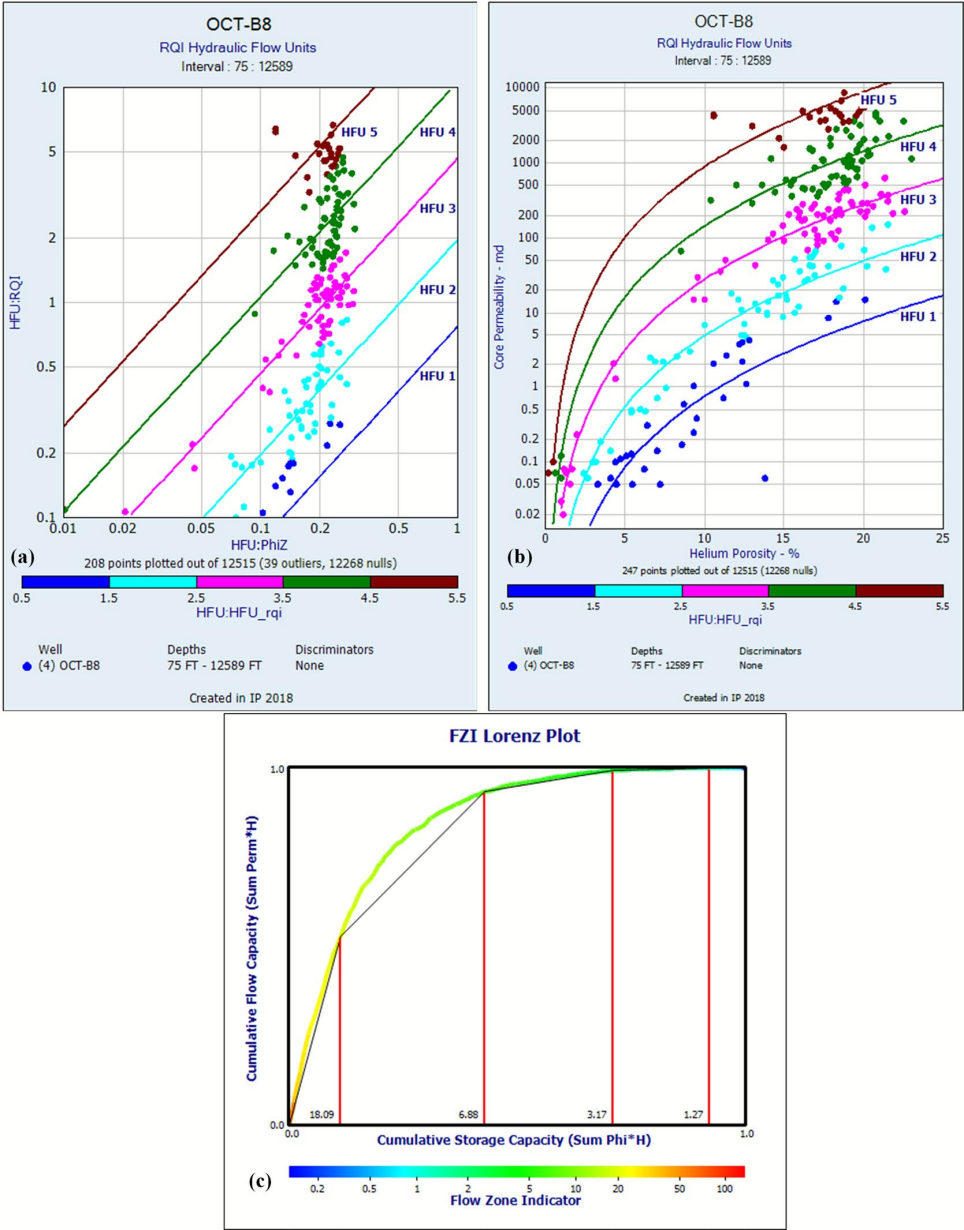
Hydraulic Flow Unit (HFU) characterization: (**a**) FZI-based clustering, (**b**) HFU-specific ϕ-k transforms, and (**c**) SML plot validating flow unit segmentation.

Building upon the validated core-log petrophysical parameters (Sect. “Well log analysis”), HFUs within the reservoir were rigorously delineated using the FZI methodology. FZI was calculated for each core sample via RQI and ϕ_z_. Samples exhibiting statistically similar FZI values were clustered, defining distinct HFUs characterized by homogeneous pore geometry and consistent fluid flow behavior. Cluster analysis of 208 core samples, based on histogram separation of calculated FZI values, revealed five statistically distinct HFUs (Fig. 14a,b), each characterized by homogeneous pore geometry and consistent fluid flow behavior. Figure 14a quantifies the functional relationship governing RQI as a function of ϕ_z_, while Fig. 14b establishes the statistically derived porosity–permeability (ϕ-k) transformation from core analysis data. This classification was corroborated by SML analysis (Fig. 14c), where cumulative flow capacity (∑k·h) versus storage capacity (∑ϕ·h) exhibited five discrete linear segments, confirming equivalent hydraulic zonation. Five dominant HFUs (HFU 1 to HFU 5) were identified within the cored interval. Each HFU is defined by a unique, statistically significant power-law relationship between (ϕ) and (k) (Fig. 14b, Table 2), derived from the fundamental FZI equation. These relationships represent optimized, unit-specific permeability transforms (Table 2).

**Table 2:** Hydraulic flow unit (HFU) permeability transform equations and statistical validation.

HFU	Equation	R^2^
HFU 1	Perm = Phi^3^ * (0.776 / (0.0314 * (1.0-Phi))) ^2^	0.72
HFU 2	Perm = Phi^3^ * (1.968 / (0.0314 * (1.0-Phi))) ^2^	0.93
HFU 3	Perm = Phi^3^ * (4.696 / (0.0314 * (1.0-Phi))) ^2^	0.97
HFU 4	Perm = Phi^3^ * (10.656 / (0.0314 * (1.0-Phi))) ^2^	0.93
HFU 5	Perm = Phi^3^ * (26.580 / (0.0314 * (1.0-Phi))) ^2^	0.91

Regression analysis yielded exceptionally strong coefficients of determination (R^2^) for all HFUs, ranging from 0.72 (HFU 1) to 0.97 (HFU 3), with HFUs 2, 4, and 5 exhibiting R^2^ values of 0.93, 0.93, and 0.91, respectively. The high R^2^ values confirm the robustness of the FZI clustering and the predictive capability of the derived equations within each flow unit (Table 2). The assigned FZI constants embedded within the permeability equations (Table 2) exhibit a systematic increase spanning nearly two orders of magnitude, from 0.776 (HFU 1) to 26.580 (HFU 5). This progression signifies a fundamental enhancement in pore-scale attributes across the HFU sequence. Lower FZI values (HFU 1) indicate less efficient pore systems characterized by smaller mean hydraulic radii, higher tortuosity, and/or unfavorable shape factors. Conversely, higher FZI values (HFU 5) denote progressively superior flow efficiency resulting from larger pore throats, lower flow path tortuosity, and more favorable pore geometry. Consequently, HFU 5 represents the highest reservoir quality facies, while HFU 1 represents the lowest quality rock within the classified spectrum.

The statistically robust HFU-specific permeability transforms (Table 2) provide a powerful predictive tool. By assigning the appropriate HFU based on log-derived properties (e.g., within electrofacies or via FZI prediction from porosity), these equations enable the accurate prediction of permeability in uncored intervals and wells using only standard porosity logs. This significantly reduces reliance on extensive coring programs and enhances the spatial characterization of reservoir deliverability. The distinct FZI values associated with each HFU directly reflect their inherent pore geometry, forming a critical foundation for predicting dynamic properties such as capillary pressure and relative permeability curves within the reservoir model.

## Discussion

This integrated study advances the characterization of pre-rift reservoirs in the October Field by synergizing multi-scale datasets to resolve uncertainties in structure, reservoir quality, and fluid flow capacity. The following discussion critically situates our findings within the context of existing literature, elucidates the diagenetic implications of our quantitative data, and highlights the novel contributions of our workflow.

### Structural compartmentalization and fault reactivation

Our seismic interpretation delineates a structural framework dominated by NW–SE trending normal faults, consistent with the regional tectonic grain of the central Gulf of Suez^1,30^.The identification of a NE-SW oriented fault, aligning with the Gulf of Aqaba trend, suggests post-Miocene reactivation, a phenomenon increasingly recognized as a critical control on compartmentalization in mature rift basins^2,7,29^. While previous 3D structural models have effectively mapped fault geometries^4,7^, our study integrates this framework with dynamic flow unit analysis. This integration reveals that these faults do not merely create structural traps but also define the boundaries of distinct hydraulic compartments, as evidenced by the preferential localization of high hydrocarbon saturation (Sₕ > 80%) within fault-bounded blocks of the Nubia Formation (Figs. 8, 11). This finding moves beyond static structural mapping and provides a direct link between faulting and reservoir performance, a crucial insight for optimizing infill drilling and secondary recovery projects.

### Reservoir quality and petrophysical superiority of the Nubia formation

The relatively higher shale content and variable reservoir quality observed in the Matulla Formation of the October Field align with regional studies of the Upper Cretaceous Wata-Matulla interval^36^. These studies link facies variability to the interplay of eustatic sea-level change and differential tectonic subsidence on the evolving rift margin^28^. This regional tectono-stratigraphic setting provides a genetic explanation for the heterogeneous mix of sandstone, shale, and carbonate facies we observe, which in turn controls the complex porosity–permeability relationships and compartmentalization. Therefore, the reservoir heterogeneity we quantify is not an isolated phenomenon but a characteristic feature of these syn-tectonic deposits, underscoring the necessity of our detailed, field-specific characterization for effective development. Our petrophysical evaluation, contextualized by this framework, confirms the Nubia Formation as the superior reservoir unit, characterized by greater net thickness (310–484 ft), lower shale volume (Vsh ≈ 5%), and higher oil saturation (up to 85%) compared to the Matulla Formation (Table 1). This aligns with regional studies that recognize the Pre-Cenomanian Nubian Sandstone as a prolific hydrocarbon-bearing interval^5,70^. However, our core-calibrated analysis provides a higher degree of certainty. For instance, whereas^70^ reported variable petrophysical parameters for the Matulla Formation in the Rabeh East Field, our study, constrained by a robust core-log porosity transform (R^2^ = 0.955), delivers precise, low-uncertainty estimates of effective porosity and hydrocarbon saturation specific to the October Field.

The determination of distinct formation water resistivity (Rw) values—0.0227 Ω·m for Matulla and 0.0582 Ω·m for Nubia—is a critical advancement. This quantitatively confirms different aquifer systems and salinities, which has significant implications for calculating original oil-in-place and planning for waterflooding, a key development strategy in the Gulf of Suez^71^. Our findings thus refine the reservoir models proposed by earlier works^9^ by incorporating fluid property variations that directly impact volumetric assessments and recovery factors.

### Novelty in hydraulic flow unit characterization and permeability prediction

A central novel contribution of this study is the rigorous application of HFU analysis using the synergistic combination of FZI clustering and SML plotting. While rock typing has been applied in the Gulf of Suez^34^, our study defines five statistically robust HFUs (R^2^ = 0.72–0.97) with unit-specific permeability transforms (Table 2). This represents a significant refinement over more generalized porosity–permeability relationships often used in the region, as evidenced by the high coefficients of determination (R^2^ = 0.72–0.97) for the unit-specific permeability transforms, which confirm the statistical robustness of the FZI clustering and its strong predictive capability.

The 34-fold range in FZI values (0.776 to 26.580) is not merely a statistical outcome but a quantitative measure of pore-system heterogeneity. This approach allows for a more accurate prediction of permeability in uncored intervals, reducing uncertainty to < 10%, a substantial improvement over traditional method. Our methodology is comparable to advanced studies in other basins^54,58^ and its successful application here demonstrates its transferability to the complex pre-rift reservoirs of the GSRB. By enabling the identification of high-flow-efficiency units (HFUs 4–5) from standard logs, this workflow provides a practical tool for targeting “sweet spots” and optimizing well placement, addressing a key challenge in mature field development.

### Implications of pore geometry and diagenetic history

Although direct petrographic data (thin sections) were not available for this study, the core-derived measurements provide powerful proxies for interpreting diagenetic controls on reservoir quality. The narrow distribution of grain density around a mean of 2.63 g/cm^3^ (Fig. 12) strongly indicates a quartz-dominated mineralogy for the Nubia Formation. This mineralogical maturity is a key depositional precursor, but it also predisposes the sandstone to porosity-reducing quartz cementation, a common diagenetic process in deeply buried siliciclastic reservoirs^72^.

The systematic variation in FZI across the five HFUs is interpreted as a direct consequence of diagenetic heterogeneity. The low-FZI units (HFUs 1–2) likely represent intervals where porosity and permeability have been significantly reduced by diagenetic processes such as mechanical compaction and extensive quartz overgrowth. In contrast, the high-FZI units (HFUs 4–5) point to intervals where reservoir quality was preserved or enhanced. This could be due to the inhibition of quartz cementation by early grain-coating chlorite, a known porosity-preserving mechanism in the Nubia Sandstone^34^, or through the creation of secondary porosity via feldspar dissolution.

The contrast between the Matulla and Nubia formations can also be interpreted diagenetically. The higher shale volume (Vsh ~ 16%) in the Matulla suggests that its reservoir quality is more strongly influenced by depositional fabric and subsequent clay diagenesis (e.g., illitization of kaolinite), which can severely reduce permeability. The cleaner, more quartzose Nubia sandstones (Vsh ~ 5%) are instead influenced primarily by quartz cementation and the potential presence of porosity-preserving chlorite coats. This inferred diagenetic framework, derived from our quantitative data, provides a causal link between mineralogy, pore-throat geometry, and the resulting flow capacity, fulfilling the objective to link reservoir quality to geological processes.

The diagenetic heterogeneity inferred from our FZI analysis is superimposed on this primary depositional template. The cleaner, more quartzose sandstones that form the high-quality reservoir facies in the Matulla Formation are likely the thick, progradational clastic units deposited during periods of increased sediment supply, a key feature of the sequence stratigraphic model for the Upper Cretaceous in this region^28^.

### Synthesis and workflow advantages

This study demonstrates that the reservoir quality and fluid distribution in the October Field’s pre-rift reservoirs are governed by a triad of factors: (1) structural segmentation creating compartments, (2) facies heterogeneity controlling porosity distribution, and (3) diagenetic overprint defining the pore-throat geometry and flow efficiency. The holistic integration of seismic, log, and core data bridges the scale gap between basin-scale fault systems and pore-scale fluid pathways.

Compared to studies that focus on a single aspect—such as structural modeling^7^, source rock evaluation^16,27^, or standalone petrophysics^21–23,70^—our workflow provides a unified, predictive model. It effectively reduces the subsurface uncertainties that hinder development in mature rift basins, offering a clear strategy for targeting undrained, high-quality compartments (high-porosity, fault-bounded Nubia sandstones within HFUs 4–5).

## Conclusion

This integrated study resolves key uncertainties in the October Field’s pre-rift reservoirs through synergistic analysis of seismic, well log, and core datasets. Key advancements include:

1. Structural Framework: Depth-contour maps and 3D models delineate four dominant faults (three NW–SE, one NE-SW) compartmentalizing the Matulla and Nubia formations, directly controlling hydrocarbon trapping and migration pathways.
2. Reservoir Superiority: The Nubia Formation emerges as the premier reservoir, exhibiting greater net thickness (310–484 ft), lower shale volume (Vsh ≈ 5%), higher oil saturation (Sh ≤ 85%), and robust flow capacity compared to the Matulla.
3. Rock Typing Breakthrough: Five HFUs were rigorously defined via FZI clustering and SML validation. HFUs 4–5 (FZI: 10.656–26.580) represent high-flow-efficiency units with favorable pore geometry (larger throats, lower tortuosity), enabling permeability prediction in uncored sections (R^2^ = 0.91–0.93) and reducing coring requirements.
4. Core-Log Integration: Core-derived grain density (ρ_g_ = 2.63 g/cm^3^) and porosity validation (R^2^ = 0.955) underpin petrophysical accuracy, critical for saturation modeling and hydrocarbon-pore-volume calculations.
5. Dynamic Implications: HFU-specific permeability–porosity transforms, and 3D property models provide a predictive foundation for dynamic simulation, waterflood optimization, and targeting undrained compartments.

This holistic workflow establishes that reservoir quality and fluid distribution are governed by the interplay of structural segmentation, facies heterogeneity, and diagenetic pore-scale attributes. Future development should prioritize high-porosity, fault-bound Nubia sandstones within HFUs 4–5, where Sh exceeds 80%. The methodologies herein are transferable to analogous rift basins globally, enhancing recovery in mature fields.

## Data Availability

Data sets generated during the current study are available from the corresponding author on reasonable request, but restrictions apply to the availability of these data.

## List of symbols

FZI: Flow zone indicator (μm)
HFU: Hydraulic flow unit (–)
Sw: Water saturation (fraction or %)
Sh: Hydrocarbon saturation (fraction or %)
ϕ: Porosity (fraction or %)
k: Permeability (mD)
ρg: Grain density (g/cm^3^)
RQI: Reservoir quality index (μm)
SML: Stratigraphic modified Lorenz plot (–)
Vsh: Shale volume (fraction or %)
